# A complex intervention to reduce hospital admissions for people living with dementia in shared-housing arrangements in Germany: results of the multicenter, cluster-randomized controlled DemWG-study

**DOI:** 10.1186/s12916-025-04090-2

**Published:** 2025-05-06

**Authors:** Julia Misonow, Karin Wolf-Ostermann, Janissa Altona, Annika Schmidt, Susanne Stiefler, Serhat Guenay, André Kratzer, Antonia Keck, Carolin Donath, Elmar Graessel

**Affiliations:** 1 Institute for Public Health and Nursing Science (IPP), University of Bremen, Bremen, Germany; 2 Competence Center for Clinical Trials Bremen (KKSB), University of Bremen, Bremen, Germany; 3 Department of Psychiatry and Psychotherapy, Center for Health Services Research in Medicine, Uniklinikum Erlangen Friedrich-Alexander-Universität Erlangen-Nürnberg (FAU), Erlangen, Germany

**Keywords:** Dementia, Cognitive dysfunction, Psychosocial intervention, Complex intervention, Hospitalization, Shared-housing arrangements, Housing for the elderly, Randomized controlled trial

## Abstract

**Background:**

People living with dementia (PlwD) have a 1.4 times higher risk of hospitalization than people living without dementia. Hospital admissions lead to negative consequences for PlwD and people living with mild cognitive impairment (PlwMCI). Housing models such as shared-housing arrangements (SHAs), which are predominantly used by PlwD, enable care-dependent people to experience daily life as ordinary as possible. However, studies are needed to show how complex non-pharmacological interventions affect hospital admissions, especially in the SHAs setting.

**Methods:**

The longitudinal, multicenter, cluster-randomized, controlled, and prospective mixed methods study from April 1, 2019, to December 31, 2022, was part of the German DemWG study and included a waitlist control group design. The multicomponent complex intervention consisted of (a) education of nursing staff in the SHAs—at the beginning of the study, (b) digital education of general practitioners—at the beginning of the study, and (c) the multimodal, psychosocial group intervention MAKS-mk + —structured application of MAKS-mk + between t0 (baseline) and t1 (after 6 months). Longitudinal data were collected at three survey times t0-t2 (t2 at another 6 months follow-up). The primary outcome parameter—hospital admission—was assessed using the nursing documentation. Poisson-models with hierarchical random effects were used for statistical analysis.

**Results:**

Nationwide, 97 SHAs with 341 residents participated at t0. Within the longitudinal observation period (12 months, t0-t2), data from 236 participants at t1 and 168 participants at t2 with mild cognitive impairment or mild to moderate dementia were evaluated. In the intention-to-treat sample, the adjusted Poisson-model showed that participants in the intervention group (IG, *n* = 201) had a significantly lower number of hospital admissions at t1 than participants in the control group (CG, *n* = 140) (*p*-value = 0.048; CI = 0.22; 0.99). Beyond t1—“open phase” of the study, no further statistically significant long-term effects of the IG could be identified (*p*-value ≤ 0.498; CI = 0.25; 1.98).

**Conclusions:**

The complex intervention significantly reduced the number of hospital admissions for PlwD and PlwMCI in the “structured phase” of DemWG. This leads to significant improvements in the nursing care and living situation for PlwD and PlwMCI. Since the intervention has been proven to have positive effects and can be easily integrated into SHAs, regular and nationwide integration into everyday care should be given greater consideration.

**Trial registration:**

ISRCTN89825211 (Registered prospectively, 16 July 2019).

## Background

Among 83 million people living in Germany [1], there are currently almost 5.0 million care-dependent people, 1.8 million people living with dementia (PlwD) [2, 3] and the estimated population-based mild cognitive impairment (MCI) prevalence is 1.5–3.7 million [4]. According to forecasts, the number of care-dependent people is expected to rise to 7.3 and the number of PlwD to 2.8 million by the year 2050 [2].

Dementia is one of the main reasons for care-dependent people to move to institutional care arrangements [5]. Since the 1990s, in addition to traditional nursing homes, shared-housing arrangements (SHAs) have developed in Germany [6]. SHAs are small home-like care environments with a maximum of twelve older care-dependent people sharing one apartment being disconnected from residential care [7]. A multi-professional network of stakeholders and service providers such as relatives, volunteers, nurses, physicians, therapists, and landlords provide person-centered care and ensure daily routines that include activities of daily living (ADLs) and promote social contacts [8–10]. In Germany, around 4000 SHAs exist and 80% of the SHAs are predominantly inhabited by PlwD and people living with mild cognitive impairment (PlwMCI) [11]. A systematic review by Speckemeier et al. on 11 housing concepts from different countries with similar care concepts indicate that innovative housing arrangements may promote social behavior, maintain activity performance, and/or positively influence emotional status compared to more traditional settings [12].

Nevertheless, a Berlin study on outcome-related evaluation of health care for people with dementia (DeWeGe-study) by Wolf-Ostermann [13] showed that within the first 4 weeks after moving into a SHA, 8.9% required inpatient hospital treatment and 6.8% required inpatient hospitalization. Another study by Wolf-Ostermann et al. on research-based quality development to improve quality of life and preventive potential in outpatient residential communities for elderly people in need of care (WGQual-study) [14] showed comparable results with 9.4% rate of hospital admission within the first 4 weeks after moving into a SHA. In general, the risk of hospital admission is 1.4 times higher in PlwD than in people living without dementia [15]. According to the study by Pinkert & Holle (2012), one third of all people with complex comorbidity are hospitalized at least once a year [15]. Given that acute hospitals are not well prepared for PlwD [16], PlwD stay 1.4 days longer in hospital than people without dementia [17]. One of the most common reasons for all hospital admission for PlwD are falls—accounting for 24% of all hospital admissions [18]. Further reasons for hospital admission are described by Thies & Bleiler [19]: syncope (circulatory collapse), falls or fractures (28%), heart disease (17%), gastrointestinal disease (9%), pneumonia (pneumonia) (6%), and delirium (acute confusion) (5%) [19]. Overlaps can be found in Prince et al.’s publication, in which it is reported that the most common reasons for hospital admission are urogenital (urinary and genital organs) infections, nutritional and metabolic disorders, head injuries, femoral neck fractures, superficial injuries, pneumonia, and acute bronchitis [20].

Negative consequences of hospitalization often include a decline in physical and cognitive functions, reduced autonomy, but also to an increased risk of falls, malnutrition, infections, delirium, or even death in hospital [21–24]. Furthermore, hospitalization of PlwD or PlwMCI can have negative effects on their relatives, nursing staff, other residents, and the entire health care system caused by higher health care costs [25, 26]. Because residents of SHAs benefit from greater social support and better monitoring compared to those living alone, this may initially lead to higher hospitalization rates in SHAs [27]. Caregivers may thus identify previously undiagnosed or untreated health problems, especially in recently transitioned residents [27]. Although hospitalizations are not always associated with negative outcomes, reducing hospital admission by considering and observing only one consistent setting with similar nursing conditions over longer observing time represents an improvement in care.

The multimodal, psychosocial MAKS® therapy has shown positive effects on care-dependent people in nursing homes and day care centers [28–30] regarding cognitive abilities, ADLs, and behavioral and psychological symptoms of dementia (BPSD) among people with cognitive impairment. However, two systematic reviews showed that a psychosocial intervention, even if multicomponent, is unlikely to be effective in reducing the risk of hospital admissions and that there is currently no established intervention to reduce the rate of hospital admission in PlwMCI or PlwD living in nursing homes or SHAs [31, 32]. For this reason, we designed a complex intervention which component directly targeting and activating PlwD and PlwMCI are based on a broad scientific basis of existing studies and literature, have been extensively researched and have been evaluated as effective and suitable for the target group [28–30, 33, 34]. The MAKS® therapy was modified and embedded in the complex intervention as a multimodal, psychosocial group intervention with motor training (“m”), cognitive exercises (“k” for the German word *kognitiv*), and exercises for fall prevention (“ + ”) for PlwD and PlwMCI named MAKS-mk +. In addition to MAKS-mk +, two components were included that focused on the education for the nursing staff and general practitioners (GPs) providing healthcare in the SHAs—as recommended for improving health care for PlwD by the World Health Organization [35] and suggested by a recent meta-analysis [36]. In summary, MAKS-mk + is intended to serve as part of the complex intervention not only by promoting cognitive exercises, increased social interaction and support, and closer monitoring by nursing staff, but also to prevent falls. In order to support fall prevention and to provide information about other reasons for hospital admissions, staff and general practitioners are trained as part of the complex intervention.

Therefore, the main objective of the DemWG study is to proof effectiveness of the complex intervention, which main part was MAKS-mk +, on reducing the number of hospital admissions for PlwD and PlwMCI in the SHAs setting.

## Methods

### Study design

As part of the DemWG study, a longitudinal, multicenter, cluster-randomized, prospective and controlled mixed methods study was conducted from April 2019 to December 2022 with a waitlist control group (CG) design taking into account the phase model of the development and evaluation of complex interventions [37, 38]. This article focuses only on the quantitative results of the primary outcome—hospital admissions.

The longitudinal data were collected at three survey times t0-t2: t0 baseline data collection before the start of the intervention, t1 after 6 months of intervention and t2 at another 6 months follow-up. The participating SHAs, who were randomly assigned to the intervention group (IG) or CG, were free to choose a start date for the intervention between June 2020 and March 2021 due to the outbreak of the SARS-CoV-2 pandemic in 2020 and resulting restrictions.

The study was approved by the Ethics Committee of the University of Bremen (Ref. 2019–18-06–3) and prospectively registered in the ISRCTN registry on August 7, 2019 (study identification number: ISRCTN89825211). For further information on the study design, see the study protocol by Kratzer et al. [39].

### Recruitment and sample size

SHAs were recruited in all federal states of Germany between July 2019 and December 2020. One hundred twenty one SHAs with a total of 1077 residents were screened for further participation. At the beginning of the study, recruitment was aimed at all SHAs in Bavaria, Berlin, Bremen, and Hamburg, where PlwMCI and PlwD lived. Due to the SARS-CoV-2 pandemic, recruitment was opened to the remaining federal states in Germany to reach a sufficient number of participants. In order to achieve the planned sample size and to recognize the additional effort associated with participating in the study, the SHAs received compensation for completing the questionnaires. After the study, the SHAs retained the complex intervention with all associated materials for further use. The principle of continuous consent was maintained throughout the study.

Regarding the primary outcome of hospital admissions, a power analysis based on experience from the WGQual study [14] was carried out prior to the end of recruitment (t0). Within the available DemWG baseline data, a 6-month hospital admission rate of 19.6% was evident. Assuming a relative reduction of another 50% [40] (i.e., from 19.6 to 9.8%), the number of participants already recruited at that time with a total of 88 SHAs and 330 PlwD and PlwMCI and an intraclass correlation coefficient (ICC) of 0.074 estimated from the data, resulted in a power of 69% for the two-sided comparison at a significance level of 5%. As a result of the intervention, no higher hospital admissions were assumed for the further course in the intervention group. This led to the assumption of a one-sided comparison at a significance level of 5% and a power of 79%. The final target sample size to achieve the desired statistical power was 80 SHAs and 330 participants.

### Eligibility of participants

As part of the screening, people living in the SHAs (with outpatient care) with MCI or mild to moderate dementia were selected on psychometric criteria. A definition of cognitive impairment according to MCI or mild to moderate dementia was performed using the Mini-Mental State Examination (MMSE) [41] and the Montreal Cognitive Assessment (MoCA) [42]. PlwD who achieved a MMSE score of 10–23 or PlwMCI who achieved a MMSE score of > 23 but a MoCA score < 24 were included—according to Book et al. [43]. In addition, informed consent from the target group has been obtained. Exclusion criteria included severe dementia (i.e., MMSE < 10) existing bedrest or permanent immobility, deafness or severe hearing impairment despite hearing aids, blindness or severe visual impairment despite visual aids, knowledge of more than one stroke, cognitive decline due to diseases other than dementia (e.g., schizophrenia or Korsakoff syndrome) medically diagnosed severe major depression, addiction, inability to communicate in German, and a planned move out of the SHA.

### Randomization and blinding

SHAs that are cared by common nursing services or common GPs were assigned to a cluster. Cluster randomization (block randomization) was performed externally by the Competence Center of Clinical Trials of the University of Bremen (KKSB) using the statistical software R, which randomly assigned the SHAs to IG or CG. The only information that was shared was the SHA code, and the localization of the SHA (federal state and rural vs. urban). The sizes of the blocks for the IG and CG were created at random. After baseline, each SHA in the IG received the complex intervention of the DemWG study. For ethical reasons, the SHAs in the CG were able to receive the complex intervention after t2. The waiting time until the complex intervention was received by the CG was 1 year in order to exclude seasonal effects that could have influenced the results. During cluster randomization, which was carried out separately for each federal state, attention was paid to a balanced distribution of IG and CG as well as a balanced distribution between urban (< 5000 inhabitants) and rural (> 5000 inhabitants) areas. After randomization, the KKSB sent the final group allocation to the headquarters study center, which then informed the participating SHAs about their assignment to the IG or CG. For organizational reasons, randomization was completed and communicated before screening.

The residents and the trained professionals could not be blinded because examining the non-pharmacological, complex intervention took the entire SHA into account. However, the data collection was conducted by nursing staff who did not carry out the MAKS-mk + intervention themselves.

### Complex intervention of the DemWG study

The complex intervention consisted of following three components:

• Component A, training of the nursing staff

• Component B, training of the GPs

• Component C, the multimodal psychosocial group intervention MAKS-mk+

Component A aimed the education and awareness-raising of people working in the SHAs using information brochures about health risks and risk situations. The brochure explained how avoidable hospital admissions for PlwD or PlwMCI in SHAs can be reduced. It provided information on risk assessment and offers options for action (e.g., maintaining the mobility of residents, adjust environmental conditions) and forward-looking planning: Advanced Care Planning. Furthermore, it provided training on medication intake such as selection, dosage, and patient medication adherence. Component A was implemented at the beginning of DemWG. At the same time as receiving Component A, the SHAs employees received a self-developed reflection and evaluation sheet to evaluate whether behavioral or knowledge changes could be conceivable.

Component B consisted of an advanced training for GPs using a digital article with examination question that was “Continuing Medical Education”-certified by the Bavarian State Medical Association on the topic of risk factors for hospitalization and on strategies for reducing and improving hospital admissions (e.g., information on medication intake such as selection, dosage, and patient medication adherence) for PlwD and PlwMCI. GPs in Germany have to proof a certain number of certified mandatory training hours respectively education lessons that can be completed by certified in-person-trainings or by accomplishing certified digital trainings with examination questions that have to be passed. Component B was implemented at the beginning of DemWG. The GPs also received a self-developed reflection and evaluation sheet. The evaluation sheet was sent to the GPs 6 months after the first invitation to participate in the training.

Component C consisted of a multimodal, psychosocial group intervention for PlwD and PlwMCI named MAKS-mk +. The entire SHA was taken into account so that residents of the SHAs without dementia or mild cognitive impairment who only have a care level could also participate. The MAKS-mk + intervention included movement and cognitive exercises in a group setting from the evidence-based MAKS® therapy. The movement exercises (“m”) included group training of the upper extremities, especially gross and fine motor skills and coordination while the cognitive exercises (“k”) included digital exercises on cognitive skills presented on a large screen in the commonly used living room of the SHAs such as remembering, recalling, language comprehension, linking, logical thinking, arithmetic, and reading that were carried out in the group of the participants. Training exercises for fall prevention (“ + ”) were implemented from the evidence-based OTAGO exercise program. The exercises included training of the lower extremities, in particular strengthening balance and muscle strength [33, 34]. According to a standardized manual, the recommendation to conduct the MAKS-mk + intervention was 5 days per week for 6 months (t0-t1). SHAs received a 4-h standardized training session and were provided with mini-PCs to use the MAKS-mk + software on a projector or TV so that MAKS-mk + could be carried out by the regular staff without additional personal resources. After 6 months (t1) until t2 (after 12 months), the SHAs were free to decide whether they wanted to continue MAKS-mk + or not; this is referred to as the “open phase” of the study.

### Instruments

Longitudinal data were collected using pseudonymized case report forms in paper form by pre-trained SHAs employees. Before the outbreak of the SARS-CoV-2 pandemic, the training took place in person and after the outbreak of the SARS-CoV-2 pandemic, the training took place online. Financial compensation was provided for completing the questionnaires. The following instruments were used.

### Primary outcome of the DemWG study: hospital admission

The primary outcome parameter—number and reason for hospital admissions—was taken from the nursing documentation. The data on the admission and discharge date and corresponding reasons for admission (divided into planned and unplanned) were documented. The number of hospital admissions at t0, t1, and t2 always referred retrospectively to the last 6 months before the respective data collection date. It can be ruled out that hospital admissions were counted before the first day of intervention. To ensure quality assurance, ad hoc telephone contacts with SHA staff and regular video consultations were carried out.

### Other measures (covariates)

Sociodemographic data (e.g., age, gender) as well as the average participation rate in the MAKS-mk + intervention of each participant within the 6 months between data collections as an approach to dose quantification, the level of care from 0 to 5, the number of residents in each SHA and the number of GPs and nursing staff, the number of home care services providing care in each SHA, and the number of permanently prescribed medication were collected from the nursing documentation. The following table shows other outcome measures for baseline characteristics and covariates with corresponding validated and reliable instruments [44–56] (see Table 1).

**Table 1:** Covariates for the primary outcome hospital admissions

Covariates	Instrument	Total score range
Activities of daily living (ADLs)	German version of the Barthel Index (BI) [49, 51]	0–100
Comorbidities	updated Charlson Comorbidity Index (CCI) [53]	0–24
Cognitive Function	MMSE [41]	0–30
	MoCA [42]	0–30
Risk for malnutrition	German version of the Mini Nutritional Assessment – Short Form (MNA®-SF) [57]	0–14

BI = higher scores indicate better performance of ADLs; Charlson Index = Updated and validated Charlson Comorbidity Index by Quan et al., higher scores indicate a higher 1-year comorbidity-related mortality rate, whereby a score of 5 is associated with an 85% 1-year mortality risk; MMSE = Mini-Mental State Examination, lower scores indicate more severe cognitive impairment, and a score between 0 and 9 indicates severe dementia; MoCA = Montreal Cognitive Assessment; higher scores indicating higher cognitive function; MNA®-SF = normal nutritional status defined as ≥ 12 points and a possible under nutritional status ≤ 11

In addition to the instruments mentioned above, secondary outcome parameters (qualitative data, among others) were collected using other instruments in the context of the DemWG study, the results of which will be published in separate publications.

### Statistical analysis of quantitative primary data collection

Participants were lost to follow-up at t1 (*n* = 105) and their scores had to be imputed via random forest imputation. The following reasons for the dropouts were given: No longer wished to participate in the study, moving out of the SHA, participants died, other. Single missing values were also imputed via iterative random forest. For the primary outcome—hospital admission—information was only provided if a corresponding hospitalization had taken place. Therefore, all those who did not provide any information in this regard were automatically classified as “no admission.” To examine differences between study dropouts and study completers, a dropout analysis was calculated. For this purpose, the baselines of the different collectives, i.e., IG with imputation vs. IG without imputation (analogous for CG) in terms of the main medical and nursing markers such as the MMSE score, the BI score, the MNA-SF score, were compared.

Due to the high number of dropouts and SHAs at t1, both a complete case analysis and an intention-to-treat (ITT) analysis were performed with imputed data and preliminary bivariate analyses were calculated to test for significant associations with the outcomes and thus identify control variables that needed to be included in the final multivariate models. Due to the already very limited sample size of 341 and the high drop-out rate, dividing the sample into further subgroups into planned and unplanned hospital admissions would further reduce the number of cases per group, reduce statistical power, and increase the risk of chance results. More robust results could therefore be better ensured by analyzing the pooled hospital admissions.

Since the numerical convergence of mixed models is not always guaranteed due to the complex correlation structure of the data, generalized estimating equations (GEE) were used. Additionally, GEE were chosen as it is a method for modeling longitudinal or cluster data and can be used for categorical or continuous responses and is easier to calculate than mixed models [58]. Due to the structure of hospital admissions as an endpoint, a GEE Poisson-model with hierarchical random effect from the nursing service level as the highest level to the resident level was used. The reason for including the nursing service level is that SHAs treated by the same nursing service are more similar in their correlation structure than SHAs treated by different nursing services.

For the dependent variable (primary outcome) hospital admissions, a basic model was calculated in regression models at t1 in which information such as the level of care at t0 and the group variable (IG/CG) were included as fixed effects. The level of care at t0 was taken as a fixed effect in all regression models, since the need for care increases with higher levels of care. It was therefore assumed that the risk of hospital admissions increases with level of care. To ensure a correct temporal order of cause and effect, separate GEE Poisson-models were used for each survey time (t1 and t2). Therefore, temporal changes in the level of care were described descriptively in the results below.

In addition to the basic model, an extended model was calculated in which all other independent t0 variables that were correlated with the respective primary outcome at t1 with a *p*-value of at least *p* < 0.10 in previously calculated bivariate correlation analyses were added as fixed effects. These include the number of GPs in the SHA, the number of nursing staff, the MNA-SF total score, the MMSE total score and the number of permanently prescribed medications. All tests were always carried out at the same significance level (*α* = 0.05). Separate GEE Poisson-models for survey time t1 and t2 were also used for the extended model. Therefore, temporal changes in the number of permanently prescribed medications were described descriptively.

The primary analysis strategy was ITT according to the CONSORT statement [59, 60]. During the SARS-CoV-2 pandemic, the focus was on ensuring primary care. This meant that component C of the complex intervention could not be carried out regularly five times a week as recommended. For this reason, the personal average weekly MAKS-mk + participation frequency (0 to 5 times per week; 0 for all CG participants) was listed descriptively. Attributable to the high dropout rate, the subsample for which no imputation of hospital admissions was necessary was analyzed as a sensitivity analysis. The ITT imputation procedure underestimated the true value of the hospital transfer rate. With a sensitivity analysis that only included cases in which no imputation of the primary outcome was necessary, the robustness of the results could be tested by using an alternative model. Due to the limited number of cases, further sensitivity analyses with the “as treated” -sample with the frequency of Maks-mk + as a variable were not performed.

The robust effect size index (RESI) of Vandekar et al. [61] was calculated as a measure of effect size. The effect size intervals proposed by Cohen [62] for the effect size—Cohen’s d—can be used as a guide to define similar ranges for the RESI: small (0.0–0.1), small-medium (0.1–0.25), and medium-large (0.25–0.40).

A type I error rate (alpha) of less than 5% was considered an indicator of statistical significance. Adjustments had to be made for multiple testing. Therefore, *p*-values were adjusted using the Benjamini–Hochberg method [63]. The fixed effects for both analysis strategies were adopted from the base and extended model. All data analyses were performed using R software.

## Results

### Description of clusters and study participants

**Fig. 1:**
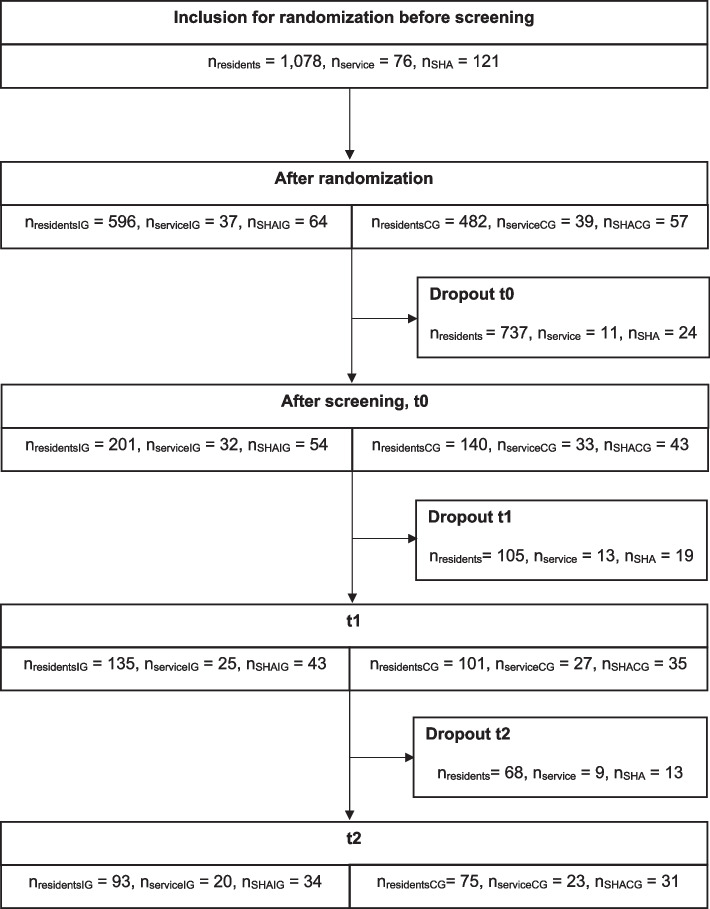
Flow chart of included residents, nursing services and SHAs at each survey time Note. nresidents = number of study participants, nservice = number of nursing services, nSHA = number of SHA, nresidentsIG = number of study participants in intervention group, nserviceIG = number of nursing services in intervention group, nSHAIG = number of SHA in intervention group, nresidentsCG = number of study participants in control group, nserviceCG = number of nursing services in control group, nSHACG = number of SHA in control group

A total of 97 SHAs (54 in IG and 43 in CG) with 341 nursing home residents and 65 home care providers were included in the study at baseline (t0). At t2 the number of SHAs still participating in the study was 65, the number of nursing home residents 168 and the number of nursing services 43. For details, see the following flow chart (Fig. 1).

The baseline characteristics of the SHAs and study participants are shown in Table 2. Most participants had care level 3 (IG = 48.8%; CG = 43.6%). The severity of participants’ cognitive impairment, as assessed by MMSE and MoCA at t0, ranged from MCI (*n* = 87, 25.5%, MMSE > 23 & MoCA < 24) to mild (*n* = 111, 32.6%, MMSE 23–18) and moderate dementia (*n* = 113, 33.1%, MMSE 17–10) to severe dementia (*n* = 30, 8.8%, MMSE < 10). In 30 individuals, cognitive impairment at t0 was consistent with severe dementia, although these individuals still had moderate (*n* = 26) or mild dementia (*n* = 4) at screening and were therefore included in the study according to the study protocol. The reason for this is that the median time interval between screening and baseline data collection respectively beginning of the complex intervention (t0) was 3 months (range: 0 to 13 months). The large difference in range can be explained by the fact that the study was interrupted due to SARS-CoV-2 pandemic and the start date of the intervention was made more flexible. Table 2 shows that there was a statistically significant difference between IG and CG regarding gender, but no difference regarding the other variables. Participants who dropped out between t0 and t1 (*n* = 105) and between t1 and t2 (*n* = 68) did not differ statistically significant from the remaining completers sample regarding the main medical and nursing markers such as the MMSE score, the BI score, and the MNA-SF score.

**Table 2:** Baseline-characteristics of the SHAs and the residents

**Variable**	**Intervention group** **(*n* = 201)**		**Control group** **(*n* = 140)**		***p*-value**
Shared-housing arrangements (SHA):					
Number of SHA staff* M* (*SD*)	11.00	(6.8)	11.50	(7.7)	0.27
Number of residents per SHA* M* (*SD*)	9.54	(2.6)	9.49	(1.9)	0.84
Participants:					
Age, *M* (*SD*)	85.40	(8.19)	83.8	(8.7)	0.08
Sex					
Female, *n* (%)	162	(80.6)	98	(70.0)	0.03
Care level, *Mdn* (*IQR*)	3.00	(1.0)	3.00	(1.0)	0.40
MMSE sum score, *M* (*SD*)	19.20	(6.02)	17.90	(6.81)	0.25
Charlson Comorbidity Index, *M (SD)*	3.27	(2.26)	3.51	(2.04)	0.45
Barthel Index, *M (SD)*	68.00	(24.9)	62.90	(26.9)	0.17

Note. *M* Mean, *SD* Standard deviation, *Mdn* Median, *IQR* Interquartile range, Care level = higher scores indicate a higher need for care, Range: 0-5; *MMSE* Mini-Mental State Examination, lower scores indicate more severe cognitive impairment, and a score between 0 and 9 indicates severe dementia, Range: 0-30; Charlson Index = Updated and validated Charlson Comorbidity Index by Quan et al., higher scores indicate a higher 1-year comorbidity-related mortality rate, Range: 0-24, whereby a score of 5 is associated with an 85% 1-year mortality risk; Barthel Index, Range: 0-100, higher scores indicate better performance of activities of daily living

Table 3 shows that at t0 the number of hospital admissions in the IG was lower than in the CG, but not significantly lower (mean in IG = 0.24 vs. mean in CG = 0.38, *p*-value = 0.089). At t1 the difference in the means increased further and also resulted in a significant difference (mean in IG = 0.23 vs. mean in CG = 0.41, *p*-value = 0.022). At t2 the difference in the means became smaller again, but the number of hospital admissions in the IG was still significantly lower than in the CG (mean in IG = 0.16 vs. mean in CG = 0.29, *p*-value = 0.049). The table also shows that the mean of care level at t1 and t2 was lower than at the survey time t0, i.e., the overall need for care at times t1 and t2 was lower than at t0. It is also clear that the number of medications intake was stable over the course of the study, despite the increased dropout rate.

**Table 3:** Unadjusted summary statistics: Number of hospital admissions, care level, and number of medications from t0 to t2

Variables	Min	Q1	Median	Mean	Q3	Max	SD	*p*-value (Adjusted *p*-value)
	**t0**							
**Number of hospital admissions**								
Intervention group	0	0	0	0.24	0	4	0.56	0.089
Control group	0	0	0	0.38	0	5	0.85	-
**Care level**	1	4	4	4.11	5	6	0.8	
**Number of medications**	0	5	7	7.47	10	23	3.52	
	**t1**							
**Number of hospital admissions**								
Intervention group	0	0	0	0.23	0	3	0.56	0.022 (0.044)
Control group	0	0	0	0.41	1	4	0.80	-
**Care level**	0	3	3	3.42	5	6	0.96	
**Number of medications**	1	5	7	7.43	10	19	3.12	
	**t2**							
**Number of hospital admissions**								
Intervention group	0	0	0	0.16	0	2	0.49	0.049 (0.049)
Control group	0	0	0	0.29	0	3	0.66	-
**Care level**	1	3	4	3.55	4	6	0.91	
**Number of medications**	1	5	7	7.33	10	15	2.81	

**Fig. 2:**
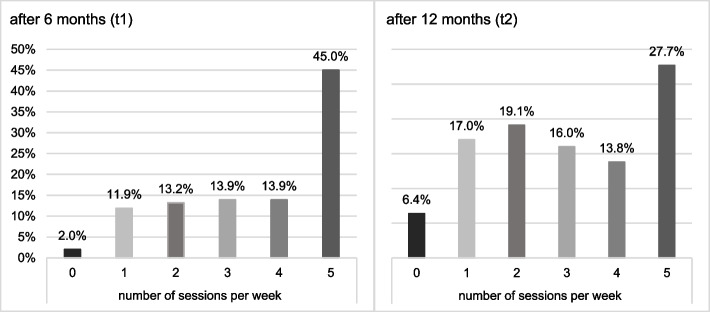
Frequency of MAKS-mk + participation in SHAs at t1, t2 in IG

At t1—at the end of the structured application of MAKS-mk +, the most common frequency of participation in the MAKS-mk + intervention was five times per week, for almost half (45%) of the IG participants with a median of 4 times a week. At t2—at the end of the “open phase,” the most common frequencies of participation in the MAKS-mk + intervention were five times per week for 27.7% and two times per week for 19.1% of IG participants with a median of 3 times a week (see Fig. 2).

### ITT analysis—basic model

Table 4 shows the estimates taken from the basic model for the influence of the individual covariates at t0 on the primary endpoint (hospital admissions) at t1. The estimates are to be understood as incidence rate ratios. The estimate for the incidence rate ratio shows that the number of hospital admissions for people in the IG is reduced compared to people in the CG at t1 (estimator = 0.41). Therefore, the intervention has a statistically significant (Benjamini–Hochberg corrected *p*-value = 0.015; CI = 0.20; 0.84) positive effect on the primary outcome for the study population (see Table 4). The computed RESI = 0.22, CI = 0; 0.43 can be considered a small- to medium-sized effect.

The power was calculated using the current number of outpatient care services and an associated estimated ICC of 0.028. With one-sided testing at the significance level of 5%, this results in a power of 72% for evidence of an effect in the primary endpoint. In the analyses for the primary endpoint, statistical significance was even found with one-sided testing at the significance level of 2.5%.

For the statistical analysis of the primary outcome at t2 with fixed effects from t0, the effect of the intervention on the primary endpoint remains, but this effect is no longer statistically significant (*p*-value ≤ 0.67; CI = 0.2; 2.79). At both t1 and t2, the level of care was not a significant predictor (see Table 4).

### ITT analysis—extended model

The basic model was extended by the fixed effects “number of GPs in the SHA,” “number of assistant physicians in the SHA,” “MNA®-SF,” “MMSE,” and “number of permanently prescribed medications” at t0. The extended model also showed a statistically significant positive effect (*p*-value = 0.048; CI = 0.22; 0.99) on hospital admission for the intervention group, as already observed in the basic model (see Table 5). However, at t2 the positive effect of the complex intervention on the primary outcome showed no statistically significant effects (*p*-value = 0.498; CI = 0.25; 1.98). Additionally, significant predictor for hospital admissions at t1 and t2 was the number of medications administered. Higher numbers of medications at t0 resulted in more hospital admissions at t1 and t2 ( see Table 5).

**Table 4:** Basic GEE-Poisson model for hospital admission with hierarchical random effect

	**Number of hospital admissions at t1**			**Number of hospital admissions at t2**		
***Predictors (at t0)***	***Estimates***	***95% CI***	***p***	***Estimates***	***95% CI***	***p***
(Intercept)	0.31	0.17–0.56	< 0.001	0.09	0.03–0.33	< 0.001
Number of hospital admissions	**1.35**	**1.04–1.76**	**0.025**	1.20	0.62–2.30	0.588
Intervention group (reference: control group)	**0.41**	**0.20–0.84**	**0.015**	0.75	0.20–2.79	0.671
Care levels 1 & 2 (reference: care level 3)	0.50	0.23–1.09	0.080	1.16	0.48–2.82	0.738
Care levels 4 & 5 (reference: care level 3)	0.70	0.37–1.34	0.284	0.52	0.17–1.59	0.251

Care level was included in the model as a categorical variable, with care level 3 as the reference category, No care level, care level 1, and care level 2 were merged into one group because the terms “no care level” and “care level 1” occurred comparatively rarely and would otherwise have led to a convergence problem of the used models.; SHA: Shared-housing arrangement; σ^2^ = variance component of the residual; τ00 nursing service = variance component of the nursing service; τ00 SHA = variance component of the SHA; ICC = intraclass correlation; nservice = number of different nursing services; nSHA = number of different SHA; nobserv = number of observations; Random effects for t1: σ^2^ = 1.92, τ00 SHA = 0.59, τ00 nursing service = 0.17, ICC = 0.28, nservice = 52, nSHA = 78, nobserv = 236; Random effects for t2: σ2 = 2.66, τ00 nursing service = 1.76, τ00 SHA = 0.37, ICC = 0.45, nservice = 43, nSHA = 65, nobserv = 168, Marginal R2/Conditional R2 = 0.025/0.459

**Table 5:** Extended GEE-Poisson model for the primary endpoint with hierarchical random effect

	**Number of hospital admissions at t1**			**Number of hospital admissions at t2**		
***Predictors (at t0)***	***Estimates***	***95% CI***	***p***	***Estimates***	***95% CI***	***p***
(Intercept)	0.05	0.01–0.45	0.007	0.00	0.00–0.02	< 0.001
Number of hospital admissions	1.24	0.94–1.64	0.129	0.98	0.49–2.00	0.965
Intervention group (reference: control group)	**0.47**	**0.22–0.99**	**0.048**	0.70	0.25–1.98	0.498
Care levels 1 & 2 (reference: care level 3)	0.53	0.24–1.15	0.109	1.29	0.55–3.05	0.558
Care levels 4 & 5 (reference: care level 3)	0.70	0.35–1.39	0.313	0.71	0.23–2.19	0.552
Number of general practitioners per participant	0.84	0.06–11.44	0.895	**79.56**	**2.59–2444.25**	**0.012**
Proportion of voluntary social year staff and volunteers per participant	0.33	0.11–1.05	0.060	0.72	0.19–2.71	0.631
MNA®-SF total score	1.03	0.89–1.19	0.701	1.29	0.98–1.69	0.068
MMSE total score	1.00	0.95–1.06	0.942	1.00	0.92–1.09	0.993
Number of permanently prescribed medications	**1.23**	**1.12–1.36**	** < 0.001**	**1.24**	**1.08–1.42**	**0.002**

Care level was included in the model as a categorical variable, with care level 3 as the reference category, No care level, care level 1, and care level 2 were merged into one group because the terms “no care level” and “care level 1” occurred comparatively rarely and would otherwise have led to a convergence problem of the used models; SHA: Shared-housing arrangement, Number of general practitioners for the SHA: Number of general practitioners who are responsible for the different residents of the relevant SHA, MNA®-SF: German version of the Mini Nutritional Assessment – Short Form, normal nutritional status defined as ≥ 12 points and a possible under nutritional status ≤ 11; MMSE: Mini-Mental State Examination, Range: 0–30, lower scores indicate higher cognitive impairment, σ^2^ = variance component of the residual; τ00 nursing service = variance component of the nursing service; τ00 SHA = variance component of the SHA; ICC = intraclass correlation; nservice = number of different nursing services; nSHA = number of different SHA; nobserv = number of observations; Random effects for t1: σ^2^ = 1.92, τ00SHA = 0.29, τ00 nursing service = 0.40, ICC = 0.26, nservice = 52, nSHA = 78, nobserv = 236; Random effects for t2: σ2 = 2.66, τ00 nursing service = 0.73, τ00 SHA = 0.00, nservice = 43, nSHA = 65, nobserv = 168, Marginal R2/Conditional R2 = 0.332/NA

### Sensitivity analysis

As a sensitivity analysis of the subsample for which no imputation of hospital admissions was necessary, the basic model showed a statistically significant effect of the intervention on hospital transfers at t1 (*p*-value = 0.011 (0.021), CI = 0.21; 0.81). The same applies to the extended model for hospital admissions at t1 (*p*-value = 0.030 (0.030), CI = 0.21; 0.93) and for the number of permanently prescribed medications at t1 (*p*-value = < 0.001 (0.021), CI = 1.11; 1.36). At time t2, no further significant effects could be demonstrated for either model. The effect size for the sensitivity analysis was a *p*-value of 0.0562 (see Table 6)

**Table 6:** Sensitivity analysis: basic and extended GEE-Poisson model for the primary endpoint without imputation with hierarchical random effect

	**Basic model - number of hospital admissions at t1**			**Extended model - number of hospital admissions at t1**		
***Predictors (at t0)***	***Estimates***	***95% CI***	***p***	***Estimates***	***95% CI***	***p***
(Intercept)	0.21	0.10–0.44	< 0.001	0.03	0.00–0.26	0.001
Number of hospital admissions	**1.35**	**1.04–1.75**	**0.023**	1.24	0.94–1.63	0.124
Intervention group (reference: control group)	**0.41**	**0.21–0.81**	**0.011 (0.021)**	**0.45**	**0.21–0.93**	**0.030 (0.030)**
Care levels 1 and 2 (reference: care level 3)	1.32	0.69–2.51	0.403	1.32	0.64–2.72	0.447
Care levels 4 and 5 (reference: care level 3)	2.14	0.56–8.20	0.269	1.79	0.45–7.10	0.410
Number of general practitioners per participant	-	-	-	1.08	0.10–12.19	0.951
Proportion of voluntary social year staff and volunteers per participant	-	-	-	0.34	0.10–1.12	0.077
MNA®-SF total score	-	-	-	1.02	0.87–1.20	0.778
MMSE total score	-	-	-	1.00	0.95–1.06	0.888
Number of permanently prescribed medications	**-**	**-**	**-**	**1.23**	**1.11–1.36**	** < 0.001**

Care level was included in the model as a categorical variable, with care level 3 as the reference category, No care level, care level 1 and care level 2 were merged into one group because the terms “no care level” and “care level 1” occurred comparatively rarely and would otherwise have led to a convergence problem of the used models; SHA: Shared-housing arrangement, Number of general practitioners for the SHA: Number of general practitioners who are responsible for the different residents of the relevant SHA, MNA®-SF: German version of the Mini Nutritional Assessment – Short Form, normal nutritional status defined as ≥ 12 points and a possible under nutritional status ≤ 11; MMSE: Mini-Mental State Examination, Range: 0–30, lower scores indicate higher cognitive impairment, σ^2^ = variance component of the residual; τ00 nursing service = variance component of the nursing service; τ00 SHA = variance component of the SHA; ICC = intraclass correlation; nservice = number of different nursing services; nSHA = number of different SHA; nobserv = number of observations; Random effects for basic model for t1: σ^2^ = 1.94, τ00 SHA = 0.60, τ00 nursing service = 0.6, ICC = 0.24, nservice = 52, nSHA = 78, nobserv = 235; Random effects for the extended model for t1: σ^2^ = 1.94, τ00SHA = 0.68, τ00 nursing service = 0.68, ICC = 0.26, nservice = 52, nSHA = 76, nobserv = 223

## Discussion

The DemWG study is the first cluster-randomized controlled trial (cRCT) to investigate the reduction of hospital admissions through a complex intervention in the SHAs setting which showed a statistically significant effect with regard to the reduction of hospital admissions in the period 6 months after baseline between IG and CG. The sample size of 341 participants from 97 SHAs is comparable to the number of participants recruited in the DeWeGe-study (572 residents from 105 SHAs) and WGQual-study (396 residents from 58 SHAs) [13, 14]. This shows that despite the lack of a register routinely collected data such as on resident numbers or levels of care, a high number of SHAs could be achieved, which is also comparable to the number of SHAs achieved in other studies.

Previous systematic reviews and meta-analyses could not report any significant results on the effectiveness of non-pharmacological interventions to reduce or influence hospital admissions. A systematic review and meta-analysis by Lee et al., including 20 RCTs and comparative studies, showed that individual psychoeducational interventions and multifactorial interventions had no effect on hospital or nursing home admissions [64]. Another systematic review with a random-effects meta-analysis by Packer et al. showed that interventions in care management, counseling/self-help (mean difference, MD, − 0.16, 95% CI = (− 0.32; 0.01)), physiotherapy/occupational therapy (mean difference, MD, − 0.16, 95% CI = (− 0.36; 0.03)), and improved GP/memory therapist consultations (mean difference, MD, − 0.14, 95% CI = (− 0.31; 0.03)) provided only small effects of shortening hospital stays. However, there was no evidence of a reduction in hospital admissions or mortality in any of the intervention categories mentioned [31]. As an explanation for the ineffectiveness of individual non-pharmacological interventions, two systematic reviews showed that a single intervention, even if consisting of multiple components, is unlikely to be effective in reducing the risk of hospital admissions [32, 33]. We conducted a complex intervention, which distinguishes us from other studies and this may be a reason for the observed effect—a significant reduction in hospital admissions in the first 6 months after beginning of the intervention. In this study phase MAKS-mk + was applied approximately as recommended—on average (median) 4 times per week.

Component C of the intervention—MAKS-mk + —could have had a crucial impact on the hospitalization rate in various ways. Firstly, a reduction in falls resulting in injury can be assumed. A trend towards this was observed in the MAKS® study in nursing homes [24]. Additionally, the complex intervention could have led to increased interaction between formal caregivers and PlwD and PlwMCI and thereby may support the reduction of hospital admissions. It is possible that the increased involvement of nursing staff with residents meant that intra-individual declines in performance and health risk situations could be registered earlier and that, against the background of the awareness raised by components A (nursing staff) and B (further training for GPs), there was a faster response to them. It implies that the interrelationship between several components is necessary and that not a single intervention but a complex intervention is crucial for reducing hospital admissions. For further developments, Component A can be modified so that, in addition to providing information materials, face-to-face or online events are also offered and carried out. Component B could also be expanded in terms of format and access form, for example through the online learning course on “MedLearning” published at the end of the study (available at the following link: https://cme.medlearning.de/medlearning/demenz_krankenhaus_rez/index.htm). The online learning course was designed to be action-oriented and expanded to include practical ideas and impulses (e.g., concrete communication strategies for dealing with dementia patients).

Another explanation could be that MAKS-mk + has a positive effect on agitation and aggression as part of BPSD—similar to the effect of MAKS® [29], since BPSD in general are reported to be a common (avoidable) reason for hospital admission [65]. The intervention could also have had a positive effect on the physical condition of the residents: through motor training, which in combination with cognitive activities leads to a reduction in the risk of falls, it contributed to this, as social support has been shown to have a positive effect on physical and mental health and should be further promoted [66]. MAKS-mk + is based on the evidence-based MAKS® therapy [28]. The implementation of MAKS® therapy in broad nursing practice, especially in day and nursing homes, was only possible by offering comprehensive, certified staff training courses by an accredited certification institute. This experience can be used to certify MAKS-mk +. Since MAKS-mk +, in contrast to MAKS® therapy, only consists of two modules and fall prevention exercises, it would also be conceivable to offer MAKS-mk + training courses over a shorter period of time and increase acceptance of participation. In order to identify which of the three components has led to a significant reduction in hospital admissions, further studies are necessary (e.g., dismantling studies, mediation analyses, or studies with an increase in MAKSmk + frequency participation).

A significant long-term effect of the intervention in the “open phase” of the study could not be shown—which firstly may be due to falling participant numbers resulting in a substantial reduction of avoidable hospital admissions [28]. Secondly, the complex intervention no longer had the impact it had in the first 6 months of the study, as the training programs had taken place some time ago and MAKS-mk + was no longer carried out as frequently. A decrease in not necessary hospital admissions of PlwD and PlwMCI is to be considered as an important result due to the negative consequences documented in the literature [67]. However, it cannot be ruled out that both the number of hospital stays and the reasons for hospital stays were influenced by the SARS-CoV-2 pandemic [68, 69].

The sensitivity analysis analyzed the subsample for which no imputation of hospital transfers was necessary. The ITT imputation procedure underestimated the true value of the hospital transfer rate. A sensitivity analysis that only included cases in which no imputation of the primary outcome was necessary may have overestimated the effect observed in the IG and CG. The true value may therefore be somewhere in between. Since the effect size of the sensitivity analysis is not significant (*p*-value = 0.0562), it can be concluded that the results are not robust and the effect of the intervention depends heavily on certain methodological decisions. Nevertheless, since the ITT and the sensitivity analysis showed a significant reduction in the outcome for hospital admissions at t1, it can still be said that the complex intervention demonstrably led to a reduction in hospital admissions.

Future studies should investigate what is needed to do so that the complex intervention can also have a long-term effect on a reduced hospital admission rate.

### Strengths

As the first cRCT to investigate hospital admissions in the SHAs setting, the DemWG study has numerous strengths. We found that this complex intervention had a small- to medium-sized effect (in terms of the RESI = 0.22, CI = 0; 0.43) [61] on hospital admissions as assessed with the nursing documentation. The number of hospital admissions can be derived as a “hard fact” from the nursing documentation. For this reason, it can be assumed that influences and distortions caused by (subjective) external assessments and changing external evaluators had no effect on hospital admissions and that good comparability in future meta-analyses is provided. Since the multimodal non-pharmacological group intervention included the entire SHAs and therefore residents and staff could not be blinded, care was taken to ensure that the external assessment questionnaires were not completed by the staff who conducted the group intervention MAKS-mk + [70]. A high number of clusters was reached (97 SHAs) being randomized with a small number of subjects (M = 3.51), resulting in small effect sample sizes of the clusters [71]. The ITT sample and multilevel models adhered to high established standards for statistical analysis [72].

### Limitations

Since there is no registration requirement for the SHAs in Germany and the SHAs therefore cannot be randomly selected from a complete database, a self-selection bias cannot be ruled out. Subsampling bias cannot be excluded when averaging the IG or CG assignment prior to screening and staff assessment. Between t0 and t1, there was a high dropout rate (*n* = 105) which can be explained by the fact that during the 6-month period many participants had already died, some no longer wanted to participate in the study, some participants moved out of the SHA or other. Nevertheless, the high dropout rate did not lead to any major problems in the power (ICC of 0.028; with one-sided testing at the significance level of 5%; power of 72%). Another limitation is the delay between randomization and the start of the intervention (0–13 months). Due to the Covid-19 pandemic that occurred in 2020, the start of the intervention could not always coincide with randomization and changes that occurred in the meantime could affect the results regardless of the intervention implemented. However, this decision was necessary to achieve a higher recruitment rate. During the SARS-CoV-2 pandemic, the focus was on ensuring primary care. This meant that component C of the complex intervention could not be carried out regularly five times a week as recommended.

## Conclusions

For the vulnerable group of PlwD and PlwMCI, hospital admissions pose severe health risks such as a decline in physical and cognitive functions, reduced autonomy, but also an increased risk of falls, malnutrition, infections, delirium, or even death in hospital [67]. Consequently, there is a need for easy-to-implement interventions for everyday care to improve the life situation of PlwD and PlwMCI in SHAs. The DemWG study showed that the number of hospital admissions could be significantly reduced through a complex intervention which includes three components: training of the nursing staff, training of the GPs, and the multimodal psychosocial group intervention MAKS-mk +. To achieve this effect, the intervention has to be applied as recommended. For this reason, the implementation of regular, everyday, non-pharmacological interventions for PlwD and PlwMCI in nursing practice in the SHAs should be promoted. By involving and further developing the complex intervention of all relevant health stakeholders such as nurses and GPs, a high quality of care for PlwD and PlwMCI can be ensured.

## Data Availability

The datasets generated during and/or analysed during the current study will be available upon request from Stephan Kloep (kloep@uni-bremen.de). Data will be available in the time interval from 12 months until 36 months after publication of the article. The data will be provided for non-commercial research purposes only to researchers with a proposal that was peer-reviewed and approved by an independent review committee. The inquiring researchers have to present an analysis plan and state the research purpose for which the data are needed, e.g. meta-analysis. Data will be available through the data warehouse of the University Bremen without any additional investigator support. The data that can be provided refer solely to the data underlying the presented results of the manuscript. They will be completely anonymized, linkage to the stored data with personal information will not be possible, thus case-specific additional information/clarification cannot be provided anymore.

## Abbreviations

ADLs: Activities of Daily Living
BI: Barthel Index
BPSD: Behavioral and psychological symptoms of dementia
CCI: Charlson Comorbidity Index
CG: Control Group
cRCT: Randomized controlled trial
GEE: Generalized Estimating Equations
ICC: Intraclass correlation
ID: Identity document
IG: Intervention Group
ITT: Intention-to-treat
KKSB: Competence Center of Clinical Trials of the University of Bremen
MAKS-mk +: Non-pharmacological multi-component group intervention with motor, cognitive and fall prevention exercises
MCI: Mild cognitive impairment
MMSE: Mini-Mental State Examination
MNA: Mini Nutritional Assessment
MNA®-SF: Mini Nutritional Assessment – Short Form
MoCA: Montreal Cognitive Assessment
PlwD: People living with dementia
PlwMCI: People living with mild cognitive impairment
SHAs: Shared-Housing Arrangements
